# Implementation of a Cervical Cancer Screening Intervention for Under- or Never-Screened Women in Ontario, Canada: Understanding the Acceptability of HPV Self-Sampling

**DOI:** 10.3390/curroncol30070497

**Published:** 2023-07-18

**Authors:** Kimberly Devotta, Mandana Vahabi, Vijayshree Prakash, Aisha K. Lofters

**Affiliations:** 1 Peter Gilgan Centre for Women’s Cancers, Women’s College Hospital, Toronto, ON M5S 1B2, Canada; 2 MAP Centre for Urban Health Solutions, St. Michael’s Hospital, Toronto, ON M5B 1T8, Canada; 3 Dalla Lana School of Public Health, University of Toronto, Toronto, ON M5T 3M7, Canada; 4 Daphne Cockwell School of Nursing, Toronto Metropolitan University (Formerly Ryerson University), Toronto, ON M5B 1Z5, Canada; 5 WECAN Research Project, Toronto Metropolitan University (Formerly Ryerson University), Toronto, ON M5B 1Z5, Canada; 6 Department of Family and Community Medicine, University of Toronto, Toronto, ON M5G 1V7, Canada

**Keywords:** cervical cancer, cervical cancer screening, HPV self-sampling, implementation, qualitative research, community champions, health equity

## Abstract

With appropriate screening, cervical cancer can be prevented. In Ontario, Canada, some groups of women have low screening rates. South Asian, Middle Eastern and North African women are particularly at risk of under-screening. Currently, cytology-based screening is used in Ontario, although the growing evidence and adoption of HPV testing for cervical screening has encouraged many jurisdictions around the world to move towards HPV testing, with the option of self-sampling. We conducted an intervention beginning in June 2018, where we recruited over 100 under- or never-screened (UNS) women who identify as South or West Asian, Middle Eastern or North African from the Greater Toronto Area, to understand the uptake and acceptability of HPV self-sampling as an alternative to a Pap test. Participants self-selected if they tried the kit or not and completed both quantitative and qualitative research activities. This paper focuses on the qualitative arm of the study, where follow-ups and five focus groups were conducted with those who tried the kit (three groups) and those who did not (two groups), as well as eight key informant interviews with community champions and others who were involved in our recruitment. We used the Consolidated Framework for Implementation Research (CFIR) to guide our data collection and analysis. Major themes around convenience, privacy and comfort came from the data as important drivers of the uptake of the intervention. The role of community champions and peers in engaging and educating UNS women, as well as having self-confidence to collect the sample, also came out as factors impacting uptake and plans for continued use. Overall, the intervention showed that HPV self-sampling is an acceptable alternative to a Pap test for some but not all UNS women in Ontario.

## 1. Introduction

With effective and timely screening, cervical cancer can be prevented. Women who are under- or never-screened (UNS) for cervical cancer are at particular risk of developing cervical cancer. More than half of all invasive cervical cancer cases happen amongst UNS women [1], making it particularly important to find effective and even innovative ways to increase participation in cervical screening. 

The Papanicolaou (Pap) test has been used to screen for cervical cancer since the 1960s in the province of Ontario [2]. By 2000, rates of screening had reached 59% of eligible women, and the Ontario Cervical Screening Program (OCSP) had been established. With the introduction of this publicly funded, organized screening program, an increase in screening participation occurred [2]. The OCSP saw some of its greatest successes in 2007–2009, with a 67% participation rate; however, since 2013, screening rates have remained stable at around 60%, well below provincial and national targets [2,3].

Further to this, our research team and others have shown that some sub-groups of women are less likely to be screened, including women living with low incomes and/or who are immigrants [4,5,6,7,8,9,10,11,12,13,14,15,16,17,18,19,20,21,22,23,24,25,26,27,28,29,30]. It has been shown that in Ontario, South Asian women (adjusted odds ratio: 0.61 [95% CI 0.59–0.64] versus non-immigrant women), followed by Middle Eastern and North African women (adjusted odds ratio: 0.68 [95% CI 0.64–0.72] versus non-immigrant women) have particularly lower rates of cervical screening [31]. This can be attributed to many multi-level factors, including cultural barriers around modesty, language barriers, discomfort with Pap tests, lack of a family physician and/or female physician, transportation and wait times [4,5,6,7,8,9,10,11,12,17,18,19,20,21,23,24,25,26,27,28,29,30]. To improve the screening rates of UNS women, it is important to find innovative methods to address these barriers that continue to persist. 

### Moving towards HPV Testing for Cervical Cancer Screening

Currently, the OCSP uses cytology-based screening (a Pap test), starting at the age of 25 years, for those with a cervix who have ever been sexually active. The province, like many other jurisdictions around the world, is in the process of moving away from cytology-based screening, given the increasing evidence around human papillomavirus (HPV) testing being more accurate for cervical cancer prevention [32]. The strong connection between HPV infections and cervical cancer has encouraged many jurisdictions to adopt HPV testing in place of Pap tests. The acceptance of HPV testing introduces the option of self-sampling for cervical cancer screening. HPV testing can be done via clinician-collected samples or devices that allow people to collect a vaginal swab themselves (i.e., self-sampling). There is strong evidence of the validity of self-sampling compared to clinician-collected samples for HPV testing, as well as of the high acceptance and positive attitudes of women [33,34,35,36,37,38].

We conducted an intervention beginning in June 2018, where we recruited over 100 under- or never-screened (UNS) women who identify as South or West Asian, Middle Eastern or North African from the Greater Toronto Area, to understand the uptake and acceptability of HPV self-sampling as an alternative to a Pap test. Participants self-selected if they tried the kit or not and completed both quantitative and qualitative research activities. In this paper, we report on findings related to the following question: what are the characteristics of the HPV self-sampling intervention design that lead to the acceptance or unacceptance of self-sampling as an alternative to a Pap test amongst UNS South Asian, West Asian, Middle Eastern and North African women? Guided by the Consolidated Framework for Implementation Research, we analyse specific constructs of the intervention to understand the acceptance of HPV self-sampling. 

## 2. Materials and Methods

### 2.1. Design

A community-based, mixed-methods design was used for this study. We have previously published a detailed protocol of our study [39], our recruitment approach [40] and the results of the reach and effectiveness (i.e., outcomes) of our intervention [41]. Here, we present the results derived from the qualitative data that go into detail to describe the implementation of our intervention. Through post-intervention focus groups, key informant interviews and follow-up calls, we describe the characteristics of the intervention, the individuals involved and the process of implementation, as well as the contextual factors of the inner- and outer-setting of the intervention. These qualitative data provide an in-depth understanding of the factors that influence women’s decision to undertake HPV self-sampling. We use the Consolidated Framework for Implementation Research (CFIR) to guide our data collection and analysis.

### 2.2. Intervention Participants and Recruitment

Using the most current OCSP guidelines for cervical screening in the province, and also informed by the recommendations for HPV testing [42,43], we recruited women aged 30–69 years who self-reported either never having had a Pap test (i.e., never screened) or having not had one in the past 4 years (i.e., under-screened). Participants also self-identified as having West or South Asian, Middle Eastern or North African ancestry. Our eligibility criteria also included living in the Greater Toronto Area of Ontario, Canada and being able to communicate and provide consent for participation in English. Women who had undergone a hysterectomy and retained their cervix were included. Women who had never been sexually active or who were pregnant were excluded, the latter because the HPV self-sampling device used in the study had not been trialed amongst pregnant women.

We engaged peers in the role of community champions for this study. We defined a community champion as a woman who identifies as West or South Asian, Middle Eastern or North African and has pre-existing connections with individuals, local community groups and organizations. We knew it would be important to move beyond traditional avenues of recruitment in order to engage women who had never been screened or were overdue. For example, we did not want to simply post flyers in healthcare centres or ask healthcare providers to make referrals, as our sample would then reflect women with a minimum level of access to care. Our recruitment approach included a variety of social spaces, such as places of worship, entertainment events and gatherings at people’s homes. It also included community organizations that had culturally specific outreach and programs, including those for newcomers.

### 2.3. Intervention

In our intervention, we offered participants the opportunity to use an HPV self-sampling mail-in kit. During recruitment, the community champions had examples of the kit that they would show potential participants. We also included an image of the device and the mailer box on the study recruitment poster. Participants who self-selected to try the kit were designated as Cohort A and received a kit in a postage-paid mailer box so they could return it at no cost to them. 

We distributed the HerSwab™ HPV self-sampling kit in this study, a class 2 medical device approved by Health Canada (MDL license 94847). The HerSwab™ is a brush-type (made of soft plastic) self-sampling device with a plastic applicator handle. Participants were provided with an instruction sheet that explained how to use the device and collect the sample. Women were asked to remove the device from the package and insert the tip of the device as depicted on the instruction sheet. After inserting the tip of the device into their vagina and rotating the handle counterclockwise 3 times to ensure the brush collected cell samples near the cervix, the tip was removed from the vagina and the brush was retracted back into the handle of the device (which acts as a sheath to avoid contaminating the sample), sealed in a plastic bag, and sent in the stamped envelope by mail for testing at our designated lab in Toronto (Mount Sinai). The sample was analyzed for high-risk HPV strains, mainly HPV 16 and HPV 18, which are known to significantly increase the risk of cervical cancer.

If participants damaged or lost their kit, or if they needed to re-take their sample at the lab’s request, they were given new kits. Those who elected not to try the kit were designated as Cohort B. All participants completed an interviewer-administered questionnaire to understand their knowledge, attitudes and practices around cervical screening. Additional open-ended questions were also asked about why they decided to choose or not choose to try HPV self-sampling. All participants received a CAD 30 honorarium for completing the survey. No additional honorarium was given for completing the kit. The study coordinator first followed up with those in Cohort A within a week of survey completion to see if they had tried the self-sampling kit. Those who had used their kit were then asked a series of questions about their experience with the device. If they had not used the self-sampling kit, they were given a reminder and followed up with again. Follow-up calls continued either until the kit was used, they no longer wanted to participate, or all contact attempts were exhausted. Participants in Cohort B were asked if the study had influenced their plans to have a Pap test; they were not asked questions about self-sampling. Participants were also randomly selected and invited to take part in one of five focus groups. People in the community who were involved or consulted with during recruitment, along with our community champions, were invited to participate in a one-on-one key informant interview over the phone.

### 2.4. Theoretical Framework

The Consolidated Framework for Implementation Research (CFIR) combines and unifies key constructs drawn from a multitude of implementation theories to create a comprehensive framework for understanding effective implementation [44]. Using the CFIR to examine the presence or absence of constructs can explain why the implementation of an intervention was or was not successful [45]. Overall, the CFIR aims to predict or explain the barriers to and facilitators of effective implementation. The CFIR assesses the contextual factors of implementation, and it recognizes that interventions are not implemented in controlled environments but rather in environments with dynamic and active forces [46]. The CFIR has five major domains, and each of the domains has its own set of constructs. In the most updated version of the CFIR, where ‘intervention’ is now referred to as ‘innovation’, Damschroder et al. [46] describe the five domains as the: (i) innovation domain (formerly named ‘intervention characteristics’), which documents the innovation being implemented; (ii) outer setting domain, in which the inner setting exists, and this can include the economic, political and social context of the implementation of the intervention; (iii) inner setting domain, in which the innovation is implemented; (iv) individual domain, including the roles and characteristics of the individuals involved in the innovation; and (v) implementation process domain, which examines the activities and strategies used to implement the innovation. The intended use of the CFIR is to collect data from people who have influence and/or power over the implementation outcomes [46]. As such, after the implementation of our HPV self-sampling intervention amongst UNS South and West Asian, Middle Eastern and North African women, we engaged participants in follow-up phone calls and focus groups, while also completing key informant interviews with community champions and others involved in recruitment.

### 2.5. Data Collection

Recruitment and survey collection took place from June 2018 to February 2020. Follow-up calls with participants were completed between June 2018 and January 2021. The five focus groups took place between September 2020 and November 2020, while the key informant interviews were conducted from February 2021 to September 2021. Questions answered during the follow-up calls were recorded in the recruitment database that was set up in Microsoft Access. All the focus groups were conducted via videoconference by both KD and VP, and they were audio recorded after all attendees had provided their informed consent to participate and be recorded. The key informant interviews were conducted by KD over the phone and were also recorded upon consent. All the audio recordings were transcribed verbatim. Questions asked during the follow-up calls, focus groups and key informant interviews were informed by the CFIR.

### 2.6. Data Analysis

Transcripts of the focus groups and key informant interviews, as well as a data output of the follow-up calls from the recruitment database, were imported and organized in NVivo 12. The data were first analyzed inductively using a reciprocal coding approach [47]. Thematic analysis was performed to organize the data into smaller analytical units [48]. KD and VP each looked at the same subset of transcripts and came up with a list of codes. They then met to discuss and compare both their coding and the list of codes. With this combined code list, they each looked at additional transcripts and later met once more to discuss new codes or any clarifications for existing codes. All the codes from both these rounds were combined and a meeting was held with AL, MV, KD and VP to discuss the interpretations of the data and the code list. AL and MV had read through the transcripts that were used to come up with the codes. After the team discussed the codes and agreed on the interpretations, KD coded the rest of the transcripts. An additional team meeting was held with AL, MV, KD and VP to discuss the final list of codes. The data were then analyzed deductively, where the codes were organized into the five CFIR domains. KD and AL completed this application of the CFIR.

## 3. Results

Our HPV self-sampling intervention recruited 108 participants, of whom 69 participants chose to try the self-sampling kit (Cohort A) and 39 participants chose not to (Cohort B). All participants completed the study survey. We have reported on the survey data and details of the intervention participants’ demographics elsewhere [41]. The average age was 46 years in Cohort A and 45 years in Cohort B. The majority of participants identified as South Asian, with most being from India or Pakistan. Almost all our participants were immigrants to Canada, with most of them immigrating in the past two decades. 

Most of our participants came from the Peel Region of Ontario, where South Asians are the largest visible minority population, making up 31.6% of the region’s population, compared to the 8.7% that South Asians make up of the overall population across Ontario [49]. 

Of the 69 participants who were in Cohort A, 64 tried the kit and 61 of them completed follow-up questions about their experience and future preferences for cervical screening. During the follow-up calls, almost all participants expressed that they would use the self-sampling kit as their preferred method of cervical screening in the future. Of the 39 in Cohort B, we were able to contact 38 of them with follow-up questions 3 months after participation. We conducted eight key informant interviews with people who were involved in the recruitment, including community champions, health promoters and people who lead programs and events in Peel Region. We selected these people based on their involvement and knowledge of the community. We also held three focus groups with participants in Cohort A and two focus groups with participants in Cohort B. The focus groups ranged from four to right participants per group, with a total of 29 participants across all 5 groups. We have summarized the demographics of the focus group participants, by cohort, in Table 1. 

We present the findings of our qualitative interviews and focus groups about our HPV self-sampling intervention, using the five domains of the CFIR. Table 2 presents a summary of these findings.

### 3.1. Domain 1: Innovation (Formerly Intervention Characteristics)

In the focus groups and key informant interviews, participants discussed how the characteristics of HPV self-sampling and our intervention design impacted the acceptability of this method as an alternative to a Pap test for cervical screening. Overall, participants commented on the acceptable characteristics of the innovation design, communication o the test results, and discomfort with collecting the sample on their own.

#### 3.1.1. Acceptable Characteristics of Innovation Design: Privacy and Convenience

Many remarked how HPV self-sampling is private and therefore more acceptable as a method for screening. In particular, collecting the sample on your own and where you feel most comfortable were key to the acceptance of this method. In one of the focus groups with women who had tried the kit, many agreed with a participant who said:

*... doing by myself, it’s so easy and so personal, so it’s private too, so I don’t have to tell anybody what I am doing, what I am testing and the results are, so it’s kind of confidential you can say… I can go in the washroom and do it by myself, I don’t have to tell my husband, not my sons, so what I’m doing, why my appointment is, why I’m going, why I’m taking, you know, leaving the house.*

[R2CAOct1]

The privacy that self-sampling provides was also discussed by the key informants as a factor encouraging acceptability:

*It’s confidential. It’s private. It’s something that [women] can do on their own and maybe not as invasive because it is just a swab, not as painful. And it may be something that [women] don’t want to share with their partner or their, you know, their other family members, so its really a more personal way of doing the test and knowing that they’re, you know, they’re doing something good for the early detection.*

[KI001]

One of the most discussed themes about self-sampling was how convenient it is. Participants commented on how much time and effort was saved by collecting the sample on your own time and in your own home:

*I feel this [is] very easy … you can do it by yourself, at home, and nobody has to go make a doctor appointment and go there …it’s a very busy life… you know, we have the time for our family not ourselves, so I find that it’s very easy, convenient and we can do easily ourselves, you know, the test*

[R3CAOct1]

Tied to privacy, convenience and the ability to complete the kit in your own home, HPV self-sampling was viewed by almost everyone who had tried it as a comfortable screening experience:

*It will make our life very, very easy rather than exposing [yourself] to someone you don’t know, especially if it’s a man if you’re uncomfortable [HPV self-sampling], it’s done by yourself so it was really good.*

[R6CASept21]

#### 3.1.2. Communication of Test Results

An important aspect of the HPV self-sampling intervention was sending the sample in for testing and the results being communicated to both the participant and their healthcare provider. For those who had tried the kit, this was expressed as both a facilitator and barrier to continued use. In one of the focus groups, a participant described how she was delayed sending in her sample because the provided mailer box was too big for the mailbox in her neighborhood. Having to go to a post office to mail it was an additional step where mailing the sample could be delayed or potentially forgotten about. 

What some participants seemed to like about HPV self-sampling was that they received the test results directly and faster than if they had to see their healthcare provider for Pap test results. As one participant described:

*… something like this is available, you get it done and then at least you save [time going to] the doctors, the family doctor’s trip plus the lab and laboratory trip, and you know the results at least faster than this…*.

[R1CASep21]

For those who used the kit, some of the administrative aspects concerning packaging and mailing back the sample were challenging. Since this device is used completely independently, the success of collecting and having the sample tested requires a very simple and streamlined approach. Some people had some challenges with this:

*I am just very forgetful and absentminded generally, so where I completed the test and I left out the form that had all the information out, so I secured everything and then I looked at the page [Laugh] and turned it around and I was like, you know, oh, I think this needs to go in [Laugh] there, so I had to re-open it.*

[R2CANov18]

#### 3.1.3. Discomfort with Collecting Sample on Their Own

The focus groups and key informant interviews also discussed what barriers exist for people to continue to use HPV self-sampling for cervical cancer screening past the timeline of our study. The level of independence for self-sampling was a barrier for some people to participate. In particular, not having someone there to reassure you that you are using the device correctly was highlighted by some.

*Am I doing it correctly? [Pause] They may not have that, the doctor or the practitioner, right there to ask questions to, like they would in a Pap. They’re not in the office, you know, doing the test, so yeah… is this going to be as good as a Pap?... is it better for me to have a Pap than do the self-sample? What are the percentages of, what is the word I’m looking for? Is a Pap better than the self-test? Exactly. Is it going to be, because the doctor is not doing it, am I going to have the proper result?*

[KI001]

### 3.2. Domain 2: Outer Setting

As discussed earlier, the outer setting is the economic, political and social context of the implementation of the intervention that may impact uptake of cancer screening. In the focus groups and key informant interviews, participants discussed access to screening and the impact of the COVID-19 pandemic on cervical screening.

#### 3.2.1. Access to Screening

Access to screening refers to how people currently experience or in the past experienced accessing cervical screening. These experiences seemed to impact the uptake and acceptability of HPV self-sampling. Our intervention offered participants an opportunity to be screened without having to experience some of the barriers present when visiting a healthcare space for a Pap test:

*Healthcare is quite complicated in Canada. We cannot get the quick service. Everything, we have to wait to get in. Even if you are in Emergency, we cannot, they will do a lot of tests and then only we can see the doctor, so it’s quite complicated here in Canada.*

[R2CANov18]

*I just don’t feel comfortable. The reason being, one, the wait times are too long, and then, when you go in, it’s, you know, they rush you out in no time.*

[R7CASep21]

The challenges experienced by some participants to have a Pap test made self-sampling even more appealing, given the autonomy it creates for screening:

*The only time I got a PAP test was when I was having my kids, as part of the prenatal checkups, and even though I am a doctor myself and my family doctor is from Pakistan as well, I somehow don’t feel comfortable bringing this up because it somehow feels as if I am challenging her … if I bring it up… she’ll be like oh, yeah, whatever, I know I think you’re good.*

[R3CANov18]

For many, their previous experiences with Pap tests made them curious or interested to try HPV self-sampling in hopes that it would be a way to be screened without the negative experiences of a Pap test. Additionally, these prior experiences also seemed to play a role in how acceptable self-sampling was once they had tried it. As one of our participants who tried self-sampling describes:

*I had a Pap test many years ago, I think probably eight or so years ago, and when I had that I wasn’t very comfortable. It was painful and I had some bleeding and I just didn’t like it. So, when my doctor kept telling me that I need to go back, I just kept avoiding because I didn’t want to go back, but when I found out about this study, I thought with, I think it’s very easy when I did it and no one’s, like, no one’s looking inside or putting anything inside and especially the big thing that they put in to open the vagina, that’s very painful… [HPV self-sampling] was easy and fast and no pain and no blood and so that’s why I just loved it*

[R3CASept21]

In Cohort B, where women chose not to use the self-sampling device, we often heard how painful many felt the Pap test to be, and in some cases, this made them not want to try any device or procedure that involved insertion:

*The Pap test, I found it very, very painful; very painful. I’ve done it in the past. For the past eight or ten years, I have not been doing it … I find it very, very painful and I think about two years ago my doctor insisted on doing it and she started and she couldn’t even complete it because I was in so much pain. I told her that I cannot take this pain. Even a slight insert, I couldn’t take it and she said it will take about two more minutes and I said I can’t even take 30 s. It’s very painful and when you showed me the HPV test, I feel, you know, that would be a little more easier and, you know, more comfortable and which we can be doing it on ourselves, but I don’t know personally that I want to do it or not, but you know my belief is that a PAP test is really, really painful. I have done it so many times, I think I would rather deliver a baby than get a PAP test done.*

[R2CBNov27]

#### 3.2.2. Impact of COVID-19 Pandemic

While recruitment for the intervention ended in December 2019, some participants were still completing the kit when the COVID-19 pandemic shut down screening and limited public interaction in March 2020. Additionally, all of our focus groups happened later that year, in the Fall of 2020. The impact of the pandemic, including access to healthcare and social distancing, came up as a theme when participants discussed satisfaction with the self-sampling device and preferences for continuing to use it for future screening. In one of the focus groups where women had tried the kit, some women explained:

*… the kit is the safest option during the COVID times because you don’t need to [make] appointments or go outside.*

[R5CAOct1]

*… right now, people are worrying [about going] to the doctor. If there is anything then they’re going. If they can get this kit and they can do it by themselves at home, so it’s really helpful, yeah.*

[R2CAOct1]

### 3.3. Domain 3: Inner Setting

An important aspect of understanding implementation is the inner social setting in which the intervention took place. Critical to understanding the uptake of self-sampling, participants discussed how the lives and experiences of South Asian women impact their decisions and actions around screening, particularly cultural barriers and awareness.

#### 3.3.1. Cultural Barriers

One participant noted a belief that only ever having one sexual partner meant that you could not get cervical cancer. She explained:

*Cervical cancer is usually transmitted if you have multiple partners, and in Asian society, like, we have one partner, your husband, our husband, so in my belief that is, this is the thing that the ladies feel safe and protected.*

[R3CAOct1]

Another key informant went on to further explain the roles women play in their families and how this may prevent them from participating in cancer screening:

*You see that in the family, you see that in the society, you see that as growing, you know as a child growing up. In the movies, look at South Asian movies, where they say that oh, you know the ideal wife is supposed to look after the house, the ideal wife is supposed to look after the family, the ideal wife is sacrificing, or you sacrifice your things for the family then you are the ideal housewife, so that glorification of women as [Pause] in that way, sometimes also contributes to all these things, that if you cannot do that then you are no good.*

[KI002]

The role of culture also came up in another key informant interview where the participant described the influence of family members in women’s decision to get screened:

*… it is not like in the Western countries, so the family and friends are always there and if they know, like, oh, you don’t have problem so why are you doing this or just leave it, just drop it, you don’t need to do that, and sometimes you cannot refuse to, if you have a spouse and even if we talk about this self-sampling, if your spouse says oh, no, no, you don’t need to do that. Let’s just drop it, so sometime you just have to accept that. The composition of family in South Asian [Pause] culture that is like [Pause] family members have influence, a lot of influence, on the other family members, yes.*

[KI006]

#### 3.3.2. Awareness

Awareness around cervical screening and HPV in particular came up as themes in the interviews and focus groups. Overall, there seemed to be a need to educate and increase awareness amongst women and others in their lives. As one participant explained:

*… it’s not just a person who needs [awareness] but also you know as a community, as a whole, that we live in, like, you know, our households, so our husbands, our brothers, our fathers, our mothers, you know, mothers play a huge role when a girl is growing up and she is educating her about many things, and if mother is aware about the importance of the screening, she will, you know, she will make sure that her daughter also does it and does it regularly and understands the importance of it, so reaching out to those [people in] particular.*

[R6CASept21]

The technology of HPV self-sampling was new and unfamiliar to almost all of the women in the study. In the focus groups, they stressed the need to have more education and effective communication around this method. One participant noted how the approach of the community champion was effective and could be used as a strategy to further implement self-sampling:

*A lot of awareness and education around [HPV self-sampling] is required. So doing info sessions in high schools for girls … [community champion] if she could reach out to one person and that one person can connect another five people and get them on the phone and talk about the issues and, you know, how it helps and how is it, how easy it is to do, I think it will reach to many people and from there they will, we will, find other ways to reach out which will be more effective and stuff like that, but in a very personable manner it’s effective … that strategy was helpful*

[R6CASept21]

Participants also talked about how a lack of acceptance and awareness amongst their healthcare providers impacted how they viewed HPV self-sampling and their plans for future use. While many participants liked and even preferred HPV self-sampling for current and future cervical screening, their interactions with their healthcare providers over this procedure in lieu of a Pap test were sometimes a negative experience. Some participants discussed how their own healthcare providers were not accepting of self-sampling:

*[HPV self-sampling is] so comfortable and listening to the answers of all the ladies said on this call, I think they are on the same page with me. It should be that simple. It is something natural and we all know this regularly, we, it will make our life very, very easy rather than exposing [yourself] to someone you don’t know, especially if it’s a man if you’re uncomfortable. Nothing like having your own and it’s done by yourself so it was really good. My experience with the doctor, of course, I was expecting that my doctor may not accept this test … my experience with the doctor was even worse. She thought like I don’t even know what test I did.*

[R6CASept21]

*For women like that, it’s a wonderful kit. It’s very easy, very easy to use, and it’s not at all painful. I recommend it but the only, I mean, [main] concern is like doctors are not still recognizing that, so at the end of the day again we had to do the [Pap] test, going to the doctor and then there is no point, so as soon as if it gets launched that would be better, otherwise the kit is very good*

[R7CASept21]

### 3.4. Domain 4: Characteristics of Individuals Involved

Critical to the implementation of our HPV self-sampling intervention are the characteristics of the individuals involved. In particular, in the focus groups and interviews, we heard about the impact of community champions and other peers as well as the confidence to collect a sample by themselves.

#### 3.4.1. Implementation Leads: Community Champions and Other Peers

The success of the intervention in engaging women to try HPV self-sampling is largely attributed to the role of the community champions. We have published a paper solely focused on this [40], although we are also presenting it here, as it is a critical part of our intervention. The community champions moved beyond the walls of healthcare to have conversations about cervical screening in many community settings. The relatability and approach of the community champions often meant that cultural barriers were acknowledged and addressed. As one of our community champions explained:

*Okay, being from the same background [Pause] makes me understand their family situation and also their approach towards screening and then to put it in a way that is more acceptable by them, like telling, giving them options which would also be in line with their South Asian thinking … I was their friend… For most women it’s the personal connection that matters.*

[KI002]

Uptake and engagement with the intervention were also successful when participants who had tried the kit used their experience to encourage others to do so, highlighting the importance of peers:

*… because first I try [HPV self-sampling] myself so, and it, and I feel this is very easy, convenient … I tried and I’m very satisfied because it’s very easy … then after that I discuss my group, this is, you know, the study group, and I think that this is very good and all the women you know those, some, they have, I mean, difficulty for a problem, some they are very shy, some they have, you know, the problem for, you know, that nobody can, you know, they will bring her to the doctor, make an appointment; all those things. You know, so there are newcomers and some of them are citizens and still they have a problem.*

[KI004]

#### 3.4.2. Confidence to Collect a Sample by Themselves

Confidence in themselves to collect the sample was also cited as one challenge during the collection and also in planning to use the device in the future. In the focus groups, we heard some women talk about being unsure of their ability to appropriately collect a sample:

*I wasn’t sure [if] I did it correctly or not, but [community champion] told me you can do only one time and then you need to put it in and then mail it, and then I mail it and then I find out that day, we got the result and everything was fine, but it, because you, I was doing first time so I wasn’t sure that I did it correct or not.*

[R3CANov18]

This was also heard during the follow-up calls when we asked women if they would try the self-sampling device the next time they were due to be screened. In most cases, women were still waiting to hear back about their results, which would also indicate if they collected and packaged the sample per the instructions. Many, for example, said they could not tell whether the sample was on the device or if it was ‘sufficient.’ One participant even suggested the device should have an indicator that tells you that you have collected the sample sufficiently. This seemed to be the most common factor for whether they would try the self-sampling device again in the future.

### 3.5. Domain 5: Implementation Process

The implementation process (the activities and strategies used) of our intervention was to have community champions engage participants through a range of groups, events and venues, occurring within and outside of healthcare. The HPV self-sampling kits were primarily distributed in person by the community champions, with some kits being mailed on rare occasions. The focus groups and key informant interviews discussed other preferred possibilities for implementing and distributing the HPV self-sampling device.

#### 3.5.1. Trusted and Engaged Healthcare Providers

Some participants believed that if people were engaged with their healthcare providers and felt comfortable enough to go to them, the providers may be a preferred source to distribute the HPV self-sampling kits. This seemed to be because of the education you would receive in this encounter and also the responsibility being shared with someone:

*The GP is the best resource because it’s, she will educate and if she wants any examination, it will be done on the spot*

[R2CAOct1]

*I think if they have a provider already, like at [healthcare organization], either a doctor or a nurse practitioner, explaining how to use it and the benefits of it so in their, one of their appointment sessions and then giving it to them. I think if people don’t have a provider already, providing one of these information sessions on how to use it properly, on, this is the number to call if you have any questions, this is the percentage of, what is the self-, is there a percentage of how effective it is with the [self-sampling]? Oh, okay, so even explaining that to them. Yeah, like all of those little details might be really helpful, and then having it distributed through even that information session.*

[KI001]

#### 3.5.2. Reminders from Government

Some participants also suggested that the HPV self-sampling kit be distributed when the provincial cancer agency sent out reminders that they were due for screening. Again, here we heard that this was also a way to share the responsibility for screening and that women would not be left on their own:

*I think the government has to be responsible for this because everyone is not going to check … so government has to give every woman if they are, if they are …, by the mail so they will check and it’s good because if every woman check, it’s, like it’s only we have [community champion] and we have Vijayshree but the government they give or check [Translate] so, it’s better, I think.*

[R1CAOct1]

#### 3.5.3. Convenient Delivery

Participants also commented on the convenience of how the self-sampling kit is delivered being a key determinant of future uptake. Suggestions to mail it out to people or have it at a pharmacy were discussed by participants.

*… by mail. It’s easy. So, everyone gets, you don’t have to go to doctor and pick up or anything. You just go in the mail, check your mail and it’s in your, it’s in your mail and just go check and just post it. It’s better, I think.*

[R2CAOct1]

*It should be so much available this kit that it should be in all the drug stores, like how they have the pregnancy test kits, you know, so that whenever we feel like. It’s not necessary what your doctor says. You have to go get it tested. It’s only to make you satisfied that you’re clear.*

[R1CASept20]

## 4. Discussion

In our study, we found that what people particularly liked about HPV self-sampling was the level of privacy, convenience and overall comfort, especially compared to a Pap test. The ability of people to collect a sample in the comfort of their home, at a time and day of the week of their choosing, not only addressed some of their concerns around shyness and discomfort with healthcare providers but also some of their concerns around access to healthcare (e.g., wait times, appointment scheduling, transportation). These findings show that for those who are UNS for these reasons, HPV self-sampling is an acceptable alternative that can be critical to addressing low screening rates.

As Ontario moves to implement HPV testing in place of Pap tests for cervical screening [32], this intervention demonstrates the potential impact self-sampling can have on addressing rates of UNS. In the coming years, this will have a particular impact, given the backlog of cancer screening and services in the province is around 1.1 million from the first year of the COVID-19 pandemic alone [50]. Innovative solutions such as self-sampling can play an important role in addressing the cervical screening backlog.

Our intervention also demonstrated the impact of effective engagement. Most participants seemed to respond well to the community champions and other peers (e.g., people who helped with recruitment, participants who tried the kit and then recommended it to later participants) and were more comfortable talking about this topic with them compared to their healthcare providers and family. Our community champions had a relatability with most of the participants along the lines of gender, ethnicity, language, migration experience and other aspects of their day-to-day lives. This meant that places in the community, beyond the walls of healthcare, were appropriately identified and engaged in an effort to have what could be uncomfortable conversations for some in comfortable settings. The impacts of this included effectively communicating and educating people around HPV and self-sampling as well as the importance of cervical screening. This also meant that they may have reached participants who may not be accessing healthcare regularly or at all. This emphasizes the need for interventions to meet people where they are to be more effective.

While the community champions were able to explain how to use the kits to participants during recruitment and follow-up calls, some issues with usability do suggest ways in which the process of HPV self-sampling could be improved, and that includes the size of the box to be mailed in and ensuring a very simple process for labelling and identifying samples. Additionally, building confidence amongst people to collect their own sample and mail it in will be needed for much greater uptake. Encouragement from healthcare providers could play a critical role in this, as some even expressed a disbelief that a sample they self-collected would be comparable to having their healthcare provider collect one.

The intervention also identified the need for effective communication and awareness amongst healthcare providers about the acceptability of HPV self-sampling for cervical screening. This suggests that in order for greater implementation to be more successful, efforts will need to be made by policymakers to increase awareness and acceptance amongst family physicians and other healthcare providers.

In addition to the community champions, our study also suggests some other acceptable ways in which people could receive the self-sampling device, now or in the future. The suggestion to tie the delivery of the kit in with the letter sent out by the provincial program on the due date for screening was suggested as a way to keep people up to date on cervical screening. Related to the issue of confidence and trusted individuals, some participants also suggested obtaining the kit directly from their healthcare providers. Overall, the ability to make screening convenient emerged as one of the biggest drivers of uptake. ‘Convenient,’ however, means different things in different scenarios. Convenient to the lifestyle of household responsibilities, culture, careers, and even the COVID-19 pandemic were key to initial use and plans for future screening.

The acceptance of HPV self-sampling as an alternative to a Pap test for cervical cancer prevention is being studied in different populations. In Hong Kong, a study amongst the most UNS age groups in the country reported a high acceptance rate and positive reception to self-sampling [51]. In England, where HPV testing has been used in their national screening programme since 2019, a 2022 study surveyed the preference for self-sampling amongst over 3600 screening-eligible women and found half of them preferred self-sampling over a clinician-collected sample, and even more so amongst UNS screening-eligible people [52]. In Australia, where the switch to HPV testing has already been made, a large randomized trial of UNS women found that HPV self-sampling increased their participation [53]. Similarly, a Danish randomized-controlled trial found that offering the opportunity to use HPV self-sampling as an alternative to cytology-based screening increased participation, with even more effectiveness with a direct mailing strategy [54].

Canadian studies have also looked at the acceptability of self-sampling [26,33,35,55,56,57]. Members of our research team conducted a qualitative study with Muslim immigrant women living in Ontario and found the majority of women reported a willingness to try HPV self-sampling and would even prefer it over provider-administered methods [58]. Additionally, some studies have involved the trialing of self-sampling [33,55,57]. For example, self-sampling kits have been tried among under-housed women in British Columbia and found to be an effective way to engage those who had never been screened [33].

## 5. Limitations

The limitations of this study are attributed to the size of our sample and how that compares to the diversity of South Asian, Middle Eastern and North African women. The majority of our participants identified as Indian and Pakistani, which is reflective of our recruitment efforts being mainly in the Peel Region. While our community champions spoke a range of languages and were able to provide assistance in Hindi, Marathi, Gujarati, Punjabi and Urdu to include more than conversational English-speaking participants, this is still not completely representative of all the languages spoken by people from South Asia, the Middle East and North Africa. Additionally, our community champions related to participants in many different ways, although employing additional community champions may have introduced more diversity into our sample by way of age and other ethnicities. Additionally, the intervention being within a study meant that for some, providing informed consent and providing personal data became a barrier to their participation. Lastly, our study interacted with participants at one point in time, and therefore, we are unable to see the impact of this intervention on continued screening.

## 6. Future Research and Conclusions

Using the CFIR, this study has demonstrated the acceptability of HPV self-sampling amongst a group of women who are under- or never-screened. We described some of the reasons why women do not initially get screened or keep up with their screening, and how HPV self-sampling begins to address some of these, including privacy, convenience and comfort. The qualitative data uncovered some of the drivers of uptake, including community engagement, access to trusted individuals, education, and self-confidence. We also discussed the role of community champions and how their particular social location places them in a position between the healthcare system and members of the public, allowing them to fill a gap that is created when people are uncomfortable or unable to have conversations about cervical screening with healthcare providers and/or family.

While we successfully engaged a group of UNS women to take and follow through with trying the self-sampling kit, the fact that 39 women were in Cohort B, as well as the fact that there were women who were eligible but declined, suggest that HPV self-sampling does not address all the barriers to screening. Additional work is needed to unpack and address these additional barriers.

Additionally, we did not collect the HPV vaccination status of participants. While vaccination is recommended for prevention and not screening, vaccination status may play a role in a woman’s decision to keep up to date with cervical screening. Future work could look at how vaccination status impacts a woman’s screening uptake and perception of risk.

These presented qualitative findings highlight important considerations for the upcoming move to HPV testing for cervical screening in Ontario and many other jurisdictions. We have learned of the important role that outreach and community engagement have in the acceptance of HPV self-sampling, and also identified the need to further develop strategies to engage UNS women.

## Figures and Tables

**Table 1:** Demographics of Focus Group Participants.

	Cohort A (*n* = 19)	Cohort B (*n* = 10)
Age (collected as age)		
30–39	6	5
40–49	7	1
50–59	6	2
60–69	0	2
Ethnic/cultural origin participants could choose more than 1 option		
Afghan	1	0
Bangladeshi	0	0
Indian	8	7
Iran	0	0
Pakistani	9	3
Sri Lankan	0	0
Turkish	1	0
Iraqi	1	0
Citizenship/immigration		
Canadian citizen by birth	0	0
Canadian citizen by naturalization	12	6
Landed immigrant/permanent resident	5	3
Refugee/refugee applicant	0	0
Other	2	1
Decade of Arrival (collected as year)		
1980–1989	0	1
1990–1999	5	1
2000–2009	6	3
2010–2019	8	5
2020–present	0	0
Do you think of yourself as:		
Heterosexual/straight	19	10
What is your relationship status right now?		
Divorced/separated	2	0
Married/common law	17	9
Single, never married	0	1
Widowed	0	0
Children		
Yes	17	8
No	2	2
Number of Children	0	
One	2	2
Two	7	3
Three	6	1
Four	2	2
Five	0	0
Self-rated English literacy (i.e., reading, writing and speaking abilities)		
Excellent	3	3
Very good	9	4
Good	5	3
Fair	2	0
Poor	0	0
Highest level of education		
Less than high school (grade 8 or less)	0	0
High school (12 grades) or equivalent	1	0
College (e.g., diploma) or university (e.g., BA, BSc) some or completed	10	7
Postgraduate (e.g., MA, PhD) some or completed	8	3
Employment Status		
Unemployed	9	5
Part-time employed	2	3
Full-time employed (minimum of 35 h/week)	7	1
Other	1	1
Approximate household annual income from all sources, after taxes		
Less than $25,000	3	1
$25,000 to $40,000	1	1
$41,000 to $60,000	5	1
$61,000 to $75,000	3	3
More than $75,000	4	3
Do not know	2	0
Prefer not to answer	1	1
Had a Pap test before		
Yes	15	9
No	4	1
Unsure	0	0
Time since last Pap test		
4 years ago	3	4
4 to 7 years ago	10	4
8 to 10 years ago	1	1
More than 10 years ago	1	0

**Table 2:** Summary of Findings Using the CFIR to Describe the Intervention.

CFIR Domain	Theme	Summary
Innovation (intervention characteristics)	Acceptable characteristics of innovation design: privacy and convenience	HPV self-sampling is seen as a private and convenient way to do cervical screening.
	Communication of test results	Test results are received directly and seemingly faster than a Pap test. However, some challenges were experienced mailing the sample in, including the size of the mailer box and remembering to include all the components.
	Discomfort with collecting sample on their own	The level of independence that comes with self-sampling meant that some women did not want to try HPV self-sampling.
Outer setting	Access to screening	HPV self-sampling seemed to be a method for participants to be screened without having to experience some of the past and current barriers in healthcare spaces, including wait times, feeling rushed by healthcare providers and discomfort of Pap test.
	Impact of COVID-19 pandemic	Decreased access to healthcare services as well as social distancing made HPV self-sampling that more appealing, as participants could complete it at a location they chose, such as their home.
Inner setting	Cultural barriers	Cultural beliefs around risk factors as well as family roles impact rates of under-screening and the choices some participants made to try or not try HPV self-sampling.
	Awareness	Increased awareness about cervical screening, including HPV self-sampling, is needed amongst women, as well as their families and friends, who play influential roles in their lives. Additionally, healthcare providers needed to be more aware and accepting of HPV self-sampling.
Characteristics of individuals involved	Implementation leads: community champions and other peers	Community champions as well as other peers who had tried HPV self-sampling were key individuals in engaging and encouraging women to try HPV self-sampling.
	Confidence to collect a sample by themselves	Self-confidence to adequately collect the sample was cited as a challenge during self-collection, with some wanting to receive their results before making comments about continued use.
Implementation process	Trusted and engaged healthcare providers	Healthcare providers that were viewed as trusted and engaged were mentioned as a preferred source to distributed HPV self-sampling, outside of the study.
	Reminders from government	Distributing the HPV self-sampling kit alongside reminders from the provincial cancer agency when people were due for screening was seen as a way to effectively implement HPV self-sampling in the future.
	Convenient delivery	Future uptake of HPV self-sampling would be further encouraged if the kit was convenient to obtain, such as via mail or a local pharmacy.

## Data Availability

The datasets generated and/or analysed during the current study are not publicly available due to maintaining the privacy and confidentiality of participants, but are available from the corresponding author on reasonable request.
